# Stochastic modeling of pneumonia transmission dynamics and implications for public health control

**DOI:** 10.3389/fpubh.2026.1798847

**Published:** 2026-05-29

**Authors:** Ali Raza, Marek Lampart, Wojciech Sumelka, Eugénio M. Rocha, Faridah Alruwaili, Emad Fadhal

**Affiliations:** 1 IT4Innovations, VSB-Technical University of Ostrava, Ostrava, Czechia; 2 Jadara University Research Center, Jadara University, Irbid, Jordan; 3 Center for Research and Development in Mathematics and Applications (CIDMA), Department of Mathematics, University of Aveiro, Aveiro, Portugal; 4 Poznan University of Technology, Institute of Structural Analysis, Poznan, Poland; 5 Department of Mathematical Sciences, College of Science, Princess Nourah bint Abdulrahman University, Riyadh, Saudi Arabia; 6 Department of Mathematics and Statistics, College of Science, King Faisal University, Al Ahsa, Saudi Arabia

**Keywords:** mathematical model, non-standard finite difference method, pneumonia disease, results, stochastic and deterministic modeling

## Abstract

**Objective:**

This study investigates the transmission dynamics of pneumonia using deterministic and stochastic SCIR compartmental models. The main objective is to examine how environmental randomness, contact-rate fluctuations, and individual-level interactions influence pneumonia persistence, extinction, and public health control outcomes.

**Methods:**

The population was divided into four epidemiological classes: susceptible, carrier, infected, and recovered individuals. A deterministic model based on ordinary differential equations was first formulated, and stochastic perturbations were then introduced through Itô stochastic differential equations to represent uncertainty in disease transmission. Theoretical analysis was conducted to establish positivity, boundedness, global existence of solutions, and extinction-persistence conditions. A stochastic reproduction threshold was derived to quantify how environmental noise modifies the deterministic threshold. Since the stochastic model has no closed-form solution, numerical simulations were performed in MATLAB R2023a using Euler-Maruyama, stochastic Euler, stochastic Runge-Kutta, and stochastic non-standard finite difference methods.

**Results:**

The stochastic model produced positive, bounded, and globally defined solutions. The derived stochastic threshold showed that environmental noise reduces the effective transmission potential of pneumonia and can lead to disease extinction even when the deterministic reproduction number predicts persistence. Sensitivity analysis indicated that increasing noise intensity lowers the effective reproduction threshold and promotes extinction, whereas higher transmission rates support disease persistence. Numerical simulations confirmed the stability of the pneumonia-free and pneumonia-present equilibria under the selected parameter settings. The stochastic non-standard finite difference method preserved positivity, boundedness, and convergence more effectively than Euler-Maruyama, stochastic Euler, and stochastic Runge-Kutta methods, particularly for larger step sizes.

**Conclusion:**

The stochastic SCIR framework provides a more realistic representation of pneumonia transmission under uncertain environmental and demographic conditions. The results show that stochastic fluctuations can qualitatively alter disease dynamics by reducing effective transmission and inducing extinction even when deterministic analysis predicts persistence. These findings highlight the importance of incorporating stochastic effects into pneumonia modeling and public health decision-making. The stochastic non-standard finite difference method offers a stable and biologically meaningful tool for simulating stochastic epidemic models.

## 1 Introduction

According to global health statistics, pneumonia accounts for a significant proportion of infectious disease–related deaths annually. The transmission patterns of the disease must be understood because they serve as the foundation for developing successful intervention methods (1). Deterministic compartmental models have been widely used to describe pneumonia transmission dynamics. Various extensions of SIR and SEIR-type models have incorporated vaccination, treatment, awareness, delay effects, and saturated incidence functions (2–17). The research establishedfor research on disease-free state existence and persistent state existence and permanent stability and system transition behavior. In (18), the authors studied co-infection modeling between *Streptococcus pneumoniae* and influenza. In (19), the authors studied a delayed SEIR model, and in (20, 21), the^1^ authors studied a mathematical model of pneumonia with genetic effects. Furthermore, (1, 22) provide an interesting extension utilizing fractional calculus. In the epidemiology of pneumococci, the mode of transmission has been found to be significantly linked to nasopharyngeal colonization with *Streptococcus pneumoniae*. This process marks the initial stage of carrying the pathogen without exhibiting any symptoms prior to developing an active infection. The carriers are asymptomatic in nature; however, they play a significant role in the process of spreading the infection among the populace. There is empirical evidence suggesting that nasopharyngeal carriers serve as reservoirs, especially in communities and households, due to the closer proximity of individuals to each other.

The deterministic models succeed at estimating typical epidemic patterns but they fail to represent unpredictable environmental changes and shifting population patterns and random patterns of human contact. Researchers solved this problem through the development of stochastic epidemic models which use three mathematical methods: stochastic differential equations and Markov chains and branching processes (26, 27). The deterministic threshold values which exist in the system will change because of the stochastic formulations which bring about extinction events. Stochastic epidemic models have received substantial research attention but their use in pneumonia transmission research with threshold changes and extinction criteria and numerical methods that maintain system structure remains rare.

The work introduces new elements through its two main contributions which include The first achievement establishes a stochastic reproduction threshold which shows how environmental noise affects persistence conditions through its environmental effects. The second achievement shows which parameter ranges enable stochastic system fluctuations to cause disease extinction while deterministic systems continue to exist. The research establishes stochastic SCIR pneumonia model which shows both positivity and global existence results. The research presents a new stochastic non-standard finite difference scheme whose biological feasibility and stability properties researchers use for comparison purposes. The research findings show how environmental changes affect pneumonia spread patterns and they provide a mathematical framework which pandemic researchers can use to examine epidemic transmission uncertainty.

The primary objective of this paper is to investigate pneumonia-like diseases using mathematical models in both stochastic and deterministic frameworks. The stochastic model is based on stochastic differential equations (SDEs), and the deterministic model is based on simple ordinary differential equations (ODEs). This is why we use a stochastic analysis, and not a deterministic analysis, as it is more realistic and representative of the actual dynamics.

Moreover, the analysis helps to understand the effect of stochastic variables on the comprehension of real-world phenomena of disease transmission, providing information for public policies. In addition, an efficient numerical method was used to analyze the outcome of stochastic differential equations and compare it with other methods in the literature, showing the cost-effectiveness of the method.

The rest of the paper's plan is as follows: Section 2 provides essential properties with the formulation of the model, while Section 3 designs a stochastic model in two ways: through transition probabilities and non-parametric perturbations. Section 4 predicts numerical methods for finding the results of given stochastic differential equations in a stochastic model. Finally, the results and conclusion are presented, along with a summary of the findings, at the end of the section.

## 2 Deterministic pneumonia model

The deterministic SCIR pneumonia model considered in this study follows the formulation introduced in (2). For completeness and to provide a foundation for the stochastic extension developed later, we briefly summarize the main qualitative properties of the deterministic system. Detailed proofs of existence, positivity, boundedness, and equilibrium analysis can be found in (2) and related references.

**Figure 1:**
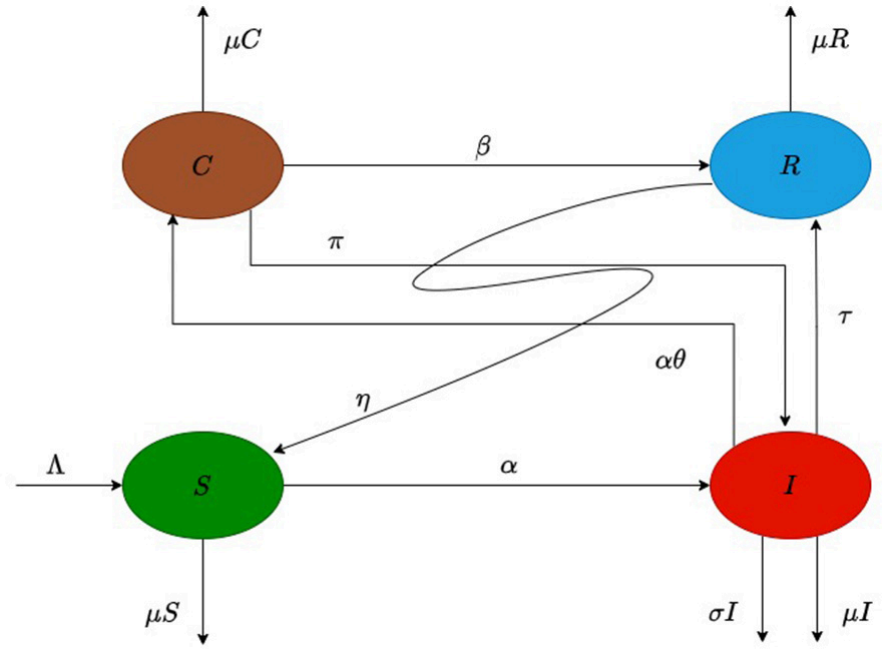
Flow chart of SCIR model of pneumonia.


The total population is divided into four epidemiological compartments: susceptible *S*(*t*), carrier *C*(*t*), infected *I*(*t*), and recovered *R*(*t*). The carrier compartment *C*(*t*) represents individuals who are colonized with *Streptococcus pneumoniae* but remain asymptomatic. These individuals may still contribute to disease transmission, although at a reduced rate compared to actively infected individuals. This distinction allows the model to capture the progression from colonization to symptomatic infection and reflects the biological pathway observed in pneumococcal diseases. The paramters described as Λ: is the birth rate of pneumonia per person from susceptible person, μ: represents natural death as per person in the population, θ: is the proportion of the infected population that becomes a carrier, σ: shows that death rate of an individual by pneumonia, β: gives recover rate of transportation per person, α: shows force of infection of pneumonia in population, τ: shows the rate of population recovers from infected pneumonia individual, η: shows that when recovering population from pneumonia become susceptible population, ω: shows that coefficient of carrier population of pneumonia, π: shows the rate as per person carrier become infected population, δ: represents pneumonia transmission rate in population, κ: contacts rate of population and *p*: maximum contact probability (See Figure 1).

$$\begin{array}{lll} \frac{dS\left(t\right)}{dt} & = & \Lambda -\left(\alpha +\mu \right)S\left(t\right)+\eta R\left(t\right) \end{array}$$ (1)

$$\begin{array}{lll} \frac{dC\left(t\right)}{dt} & = & \alpha \theta S\left(t\right)-K_{a}C\left(t\right) \end{array}$$ (2)

$$\begin{array}{lll} \frac{dI\left(t\right)}{dt} & = & \alpha \left(1-\theta \right)S\left(t\right)+\pi C\left(t\right)-K_{b}I\left(t\right) \end{array}$$ (3)

$$\begin{array}{lll} \frac{dR\left(t\right)}{dt} & = & \beta C\left(t\right)+\tau I\left(t\right)-\left(\mu +\eta \right)R\left(t\right). \end{array}$$ (4)

Where α represents the force of infection and has value $\alpha =\delta \left(\frac{I+\omega C\left(t\right)}{N}\right)$ and δ = κ*p*,


$$\begin{array}{lll} K_{a} & = & \left(\mu +\beta +\pi \right)\text{and} \end{array}$$

$$\begin{array}{lll} K_{b} & = & \tau +\mu +\sigma \frac{dN\left(t\right)}{dt}=\Lambda -\mu N-\sigma I. \end{array}$$ (5)

The initial condition is S (0) = *S*_0_, C (0) = *C*_0_I (0) = *I*_0_ R (0) = *R*_0_, N (0) = *N*_0_. The feasible region of a model $\text{Ψ}=\left\{\left(S,C,I,R\right)\epsilon {ℜ}_{+}^{4}:0\leq N\leq \frac{\Lambda }{\mu }\right\}$ where *N* = *S*+*C*+*I*+*R*.

If $\frac{dX_{i}}{dt}>0,$ at *X*_*i*_ = 0, *f*_*i*_ are Lipschitz continuous, and the initial conditions are all positive, then *X*_*i*_≥0.

Lemma 1 (Positivity of Solutions)

Lemma 1. The solutions of system (1)–(4) remain non-negative for all *t*≥0 provided non-negative initial conditions are assumed.

Proof. The proof follows standard arguments based on the invariance of the positive orthant and can be found in (2).

Lemma 2 (Boundedness and Invariant Region)

Lemma 2. All solutions of system (1)–(4) with non-negative initial conditions remain bounded in the feasible region


$$\begin{array}{l} \Omega =\left\{\left(S,C,I,R\right)\in {\mathbb{R}}_{+}^{4}:N\left(t\right)\leq \Lambda /\mu \right\}. \end{array}$$

Proof. The argument is based on the total population equation and comparison principles; see (2) for details.

### 2.1 Model equilibrium

The equilibria of the deterministic system (1)–(4) are obtained by setting


$$\begin{array}{l} \frac{dS}{dt}=\frac{dC}{dt}=\frac{dI}{dt}=\frac{dR}{dt}=0. \end{array}$$

#### 2.1.1 Pneumonia-free equilibrium (PFE)

The pneumonia-free equilibrium corresponds to the absence of carriers and infected individuals in the population. It is given by


$$\begin{array}{l} E_{0}=\left(\frac{\Lambda }{\mu },0,0,0\right). \end{array}$$

This equilibrium always exists and represents a disease-free population maintained solely by demographic processes.

#### 2.1.2 Pneumonia-present equilibrium (PPE)

When pneumonia persists in the population, the system admits an endemic (pneumonia-present) equilibrium


$$\begin{array}{l} E_{1}=\left(S^{*},C^{*},I^{*},R^{*}\right) \end{array}$$

where all components are strictly positive provided that *ℛ*_0_>1.

The equilibrium values are given by


$$\begin{array}{lll} S^{*} & = & \frac{1}{R_{0}},\\ C^{*} & = & \frac{\theta K_{b}\left(\mu +\eta \right)\left(\Lambda R_{0}-\mu \right)}{R_{0}\left[K_{b}\left(K_{a}\left(\mu +\eta \right)-\eta \theta \beta \right)-\eta \tau \left(K_{a}\left(1-\theta \right)+\pi \theta \right)\right]},\\ I^{*} & = & \frac{\left(\Lambda R_{0}-\mu \right)\left(K_{a}\left(1-\theta \right)+\pi \theta \right)\left(\mu +\eta \right)}{R_{0}\left[K_{b}\left(K_{a}\left(\mu +\eta \right)-\eta \theta \beta \right)-\eta \tau \left(K_{a}\left(1-\theta \right)+\pi \theta \right)\right]}, \end{array}$$

$$\begin{array}{l} R^{*}=\frac{\beta \theta K_{b}\left(\Lambda R_{0}-\mu \right)+\tau \left(\Lambda R_{0}-\mu \right)\left(K_{a}\left(1-\theta \right)+\pi \theta \right)}{R_{0}\left[K_{b}\left(K_{a}\left(\mu +\eta \right)-\eta \theta \beta \right)-\eta \tau \left(K_{a}\left(1-\theta \right)+\pi \theta \right)\right]-\sigma \left(K_{a}\left(1-\theta \right)+\pi \theta \right)\left(\mu +\eta \right)}. \end{array}$$

The endemic equilibrium exists and is biologically meaningful if all denominators are positive and Λℛ_0_> μ.

#### 2.1.3 Basic reproduction number

Using the next-generation matrix approach as described in (2), the basic reproduction number is given


$$\begin{array}{l} {ℛ}_{0}=\frac{\delta \left[K_{a}\left(1-\theta \right)+\theta \left(\omega K_{b}+\pi \right)\right]}{K_{a}K_{b}}. \end{array}$$

The quantity ℛ_0_ measures the average number of secondary infections generated by a single infectious individual introduced into a fully susceptible population.

- If ℛ_0_ < 1, the pneumonia-free equilibrium *E*_0_ is expected to be stable.
- If ℛ_0_>1, the pneumonia-present equilibrium *E*_1_ exists and the disease persists in the population.

It should be emphasized that the deterministic analysis presented above is not the primary contribution of this work. Rather, it serves as a reference framework for the stochastic extension developed in Section 3, where environmental perturbations fundamentally modify the persistence and extinction dynamics.

## 3 Stochastic pneumonia model

Deterministic epidemic models explain the standard spread pattern of diseases. Actual spread patterns of diseases show variations because of changing environmental conditions and human behavior and unpredictable contact patterns. We implement Itô stochastic differential equations (SDEs) to create a stochastic model that extends the deterministic SCIR model through uncertainty representation. The field of stochastic epidemic modeling enables researchers to study how demographic and environmental changes impact disease spread patterns (Raza et al. (23); Raza et al. (24)). Stochastic perturbations in this system describe how transmission rates and recovery times and demographic relationships experience random changes.

Biological Interpretation of Stochastic Terms:

In the system (6), stochastic perturbations appear in a multiplicative way, for example, σ_1_*SdB*(*t*). These terms model the environmental stochasticity and contact rate fluctuation that increase linearly with population size. There is an order of magnitude more absolute variability in transmission events in larger populations, so we presume noise strength to be directly proportional to the compartment size.

This multiplicative structure ensures:

Preservation of non-negativity,
Biologically meaningful proportional fluctuations,
Correction of meaningful transmission thresholds by noise-induced drift.

Mathematical Framework and Objectives:

The stochastic model is formulated as a system of Itô stochastic differential equations driven by independent standard Brownian motions. The main objectives of this stochastic analysis are:

1. To establish positivity and global existence of solutions.
2. To derive a stochastic reproduction threshold.
3. To identify conditions for disease extinction and persistence under environmental noise.
4. To compare stochastic and deterministic threshold behavior.

$$\begin{array}{l} \begin{array}{l} \begin{array}{l} dS=(\Lambda -(\alpha +\mu )S+\eta R)dt+\sigma_{1}SdB(t)\\ dC=(\alpha \theta S-(\mu +\beta +\pi )C)dt+\sigma_{2}CdB(t) \end{array} \end{array}\\ \begin{array}{l} \begin{array}{l} dI=(\alpha (1-\theta )S+\pi C-(\tau +\mu +\sigma )I)dt+\sigma_{3}IdB(t)\\ dR=(\beta C+\tau I-(\mu +\eta )R)dt+\sigma_{4}RdB(t)\;\\ where\alpha =\delta (\frac{I+\omega C}{N})and\delta =\kappa p \end{array} \end{array} \end{array}\}$$ (6)

For theoretical analysis, we assume general positive initial conditions


$$\begin{array}{l} N\left(0\right)={\left(S\left(0\right),C\left(0\right),I\left(0\right),R\left(0\right)\right)}^{T}\in {\mathbb{R}}_{+}^{4}. \end{array}$$

Specific numerical values used in simulations are introduced later in the Numerical Results section. The extinction and persistence conditions derived below depend only on model parameters and are independent of particular initial data, provided they are positive.

### 3.1 Epidemiological interpretation of stochastic perturbations

In system (6), stochastic perturbations are introduced in multiplicative form, for example σ_1_*SdB*_1_(*t*). This choice reflects environmental variability and contact-rate fluctuations that scale proportionally with population size.

From an epidemiological perspective, larger populations experience greater absolute variability in contacts, transmission events, and demographic processes. Therefore, modeling noise intensity as proportional to compartment size is biologically reasonable and commonly adopted in stochastic epidemic models [see (23)].

The multiplicative noise structure ensures:

1. Preservation of non-negativity of solutions.
2. Proportional environmental fluctuations.
3. Reduction of effective transmission potential through noise-induced drift correction.

Thus, the stochastic perturbation is not *ad hoc* but follows standard approaches in stochastic epidemic modeling.

#### 3.1.1 Positivity and boundedness of the stochastic model

Let


$$\begin{array}{l} U\left(t\right)={\left(S\left(t\right),C\left(t\right),I\left(t\right),R\left(t\right)\right)}^{⊤} \end{array}$$

denote the state vector of the stochastic pneumonia model, and define the Euclidean norm


$$\begin{array}{l} ∥U\left(t\right)∥=\sqrt{S^{2}\left(t\right)+C^{2}\left(t\right)+I^{2}\left(t\right)+R^{2}\left(t\right)} \end{array}$$ (7)

The stochastic system (6) can be written in the compact Itô form


$$\begin{array}{l} dU\left(t\right)=H\left(U,t\right)dt+K\left(U,t\right)dB\left(t\right), \end{array}$$ (8)

where *H*(*U, t*)represents the drift vector, *K*(*U, t*)is the diffusion matrix, and *B*(*t*)is a standard Brownian motion.

Let $C^{2,1}\left({\mathbb{R}}^{4}\times \left(0,\infty \right),{\mathbb{R}}_{+}\right)$ denote the class of non-negative functions that are twice continuously differentiable in *U* and once differentiable in *t*. For any *V*∈*C*^2, 1^, define the infinitesimal generator *L* by


$$\begin{array}{l} LV=V_{t}+V_{U}H+\frac{1}{2}Tr\left(K^{⊤}V_{UU}K\right). \end{array}$$

***Theorem 3*** *(Positivity and Global Existence)*

For any initial condition


$$\begin{array}{l} \left(S\left(0\right),C\left(0\right),I\left(0\right),R\left(0\right)\right)\in {\mathbb{R}}_{+}^{4}, \end{array}$$

system (6) admits a unique global solution


$$\begin{array}{l} \left(S\left(t\right),C\left(t\right),I\left(t\right),R\left(t\right)\right)\in {\mathbb{R}}_{+}^{4}\;forallt\geq 0 \end{array}$$

almost surely.

***Proof*. **Since the drift and diffusion coefficients of system (6) are locally Lipschitz continuous and satisfy linear growth conditions, standard results from stochastic differential equation theory [see (24)] guarantee existence and uniqueness of a local solution up to an explosion time **τ**_**e**_.

To prove global existence and positivity, define for *n*≥*n*_0_ the stopping time


$$\begin{array}{l} \tau_{n}=\inf\{t\geq 0:S\left(t\right)\notin \left(1/n,n\right)\;\text{or}\\ \text{C}\left(t\right)\notin \left(1/n,n\right)\;\text{or}I\left(t\right)\notin \left(1/n,n\right)\;\text{or}R\left(t\right)\notin \left(1/n,n\right)\}. \end{array}$$ (9)

The stopping time is introduced to localize the solution and prevent possible explosion of trajectories. As *karrow∞*, the stopping times form an increasing sequence, and proving that their limit is infinite ensures global existence of solutions.

Clearly, τ_*n*_ is non-decreasing and


$$\begin{array}{l} \begin{array}{c} \tau_{\infty }=\underset{narrow\infty }{\lim}\tau_{n}\leq \tau_{e}. \end{array} \end{array}$$ (10)

Consider the Lyapunov function $V:{\mathbb{R}}_{+}^{4}arrow{\mathbb{R}}_{+}$ defined by


$$\begin{array}{l} \begin{array}{c} V\left(S,C,I,R\right)=\underset{x\in \left\{S,C,I,R\right\}}{\sum }\left(x-1-\ln\;x\right). \end{array} \end{array}$$ (11)

This function is non-negative and radially unbounded on ${\mathbb{R}}_{+}^{4}$. Applying Itô's formula to *V* along solutions of system (6) yields


$$\begin{array}{l} dV\left(U\left(t\right)\right)=LV\left(U\left(t\right)\right)dt+\sum_{i=1}^{4}\sigma_{i}\left(1-x_{i}^{-1}\right)x_{i}dB_{i}\left(t\right),\\ where\;\;x_{i}\in \left\{S,C,I,R\right\}. \end{array}$$

Using standard inequalities and the positivity of the state variables, we obtain

*LV*(*U*(*t*)) ≤ *N*, for some positive constant


$$\begin{array}{l} N=\Lambda -4\mu +\sigma +\frac{1}{2}\left(\sigma_{1}^{2}+\sigma_{2}^{2}+\sigma_{3}^{2}+\sigma_{4}^{2}\right). \end{array}$$

Hence,


$$\begin{array}{l} \begin{array}{c} dV\left(U\left(t\right)\right)\leq Ndt+\overset{4}{\underset{i=1}{\sum }}\sigma_{i}x_{i}\left(t\right)dB_{i}\left(t\right). \end{array} \end{array}$$ (12)

Integrating from 0 to τ_*n*_∧*T* and taking expectations gives


$$\begin{array}{l} \begin{array}{c} EV\left(U\left(\tau_{n}\wedge T\right)\right)\leq V\left(U\left(0\right)\right)+NT. \end{array} \end{array}$$ (13)

On the event {τ_*n*_ ≤ *T*}, at least one component of *U*(τ_*n*_) equals either *n* or 1/*n*, implying


$$V(U(\tau_{n}))\geq \min\{n-1-\ln\;n,1/n-1-\ln(1/n)\;\}.$$

Letting *narrow∞* leads to a contradiction unless


$$\mathbb{P}\left(\tau_{\infty }\leq T\right)=0.$$

Since *T*>0 is arbitrary, it follows that


$$\begin{array}{l} \tau_{\infty }=\infty \;\text{almost}\text{surely}. \end{array}$$

Therefore, the solution exists globally and remains in ${\mathbb{R}}_{+}^{4}$ with probability one.

#### 3.1.2 Persistence and extinction

Let *B*(*t*) be a standard Brownian motion defined on a complete probability space, and let *I*(*t*) denote the infected population governed by the stochastic differential equation


$$\begin{array}{l} dI\left(t\right)=H\left(I\left(t\right),t\right)dt+K\left(I\left(t\right),t\right)dB\left(t\right), \end{array}$$

where *H* and *K* represent the drift and diffusion coefficients, respectively.

For system (6), the infected compartment satisfies


$$\begin{array}{ll} dI\left(t\right) & =\left[\alpha \left(1-\theta \right)S\left(t\right)+\pi C\left(t\right)-\left(\tau +\mu +\sigma \right)I\left(t\right)\right]dt \end{array}$$

$$\begin{array}{l} +\;\;\sigma_{3}I\left(t\right)dB\left(t\right). \end{array}$$ (14)

### 3.2 Stochastic reproduction number

We introduce the **stochastic reproduction number**


$$\begin{array}{l} \begin{array}{c} {ℛ}_{0}^{S}={ℛ}_{0}-\frac{\sigma_{3}^{2}}{2\left(\tau +\mu +\sigma \right)}, \end{array} \end{array}$$ (15)

which accounts for the reduction in transmission potential due to environmental noise.

It should be noted that the form of the stochastic reproduction number follows standard results in stochastic epidemic modeling derived using Itô calculus. The novelty in this work lies not in the derivation itself, but in its application to the SCIR pneumonia model and its interpretation in the presence of carrier-mediated transmission dynamics.

***Theorem 4 (Extinction of the Disease)***

If


$$R_{0}^{S}<1,$$

then the infected population *I*(*t*) of system (6) tends to zero exponentially almost surely, i.e.,


$$\begin{array}{l} \underset{tarrow\infty }{\lim I\left(t\right)}=0\;a.s. \end{array}$$

***Proof***

Applying Itô's formula to the function


$$\begin{array}{l} f\left(I\right)=\ln\;I, \end{array}$$

we obtain


$$\begin{array}{l} d\left(\ln\;I\left(t\right)\right)=\frac{1}{I\left(t\right)}dI\left(t\right)-\frac{1}{2}\frac{1}{I^{2}\left(t\right)}\left(\sigma_{3}^{2}I^{2}\left(t\right)\right)dt. \end{array}$$

Substituting from Equation (20) yields


$$\begin{array}{l} d\left(\ln\;I\left(t\right)\right)\\ =\left[\frac{\alpha \left(1-\theta \right)S\left(t\right)+\pi C\left(t\right)}{I\left(t\right)}-\left(\tau +\mu +\sigma \right)-\frac{\sigma_{3}^{2}}{2}\right]dt+\sigma_{3}dB\left(t\right). \end{array}$$

Using the boundedness of solutions established in Theorem 3 and the fact that


$$\begin{array}{l} S\left(t\right)\leq \frac{\Lambda }{\mu },C\left(t\right)\leq \frac{\Lambda }{\mu }, \end{array}$$

we obtain


$$\begin{array}{l} \frac{\alpha \left(1-\theta \right)S\left(t\right)+\pi C\left(t\right)}{I\left(t\right)}\leq \left(\tau +\mu +\sigma \right){ℛ}_{0}. \end{array}$$

Thus,


$$\begin{array}{l} d\left(\ln\;I\left(t\right)\right)\leq \left(\tau +\mu +\sigma \right)\left({ℛ}_{0}^{S}-1\right)dt+\sigma_{3}dB\left(t\right). \end{array}$$

Integrating from 0 to *t* gives


$$\begin{array}{l} \ln\;I\left(t\right)\leq \ln\;I\left(0\right)+\left(\tau +\mu +\sigma \right)\left({ℛ}_{0}^{S}-1\right)t+\sigma_{3}B\left(t\right). \end{array}$$

Dividing by *t* and letting *tarrow∞*, we use the strong law of large numbers for Brownian motion:


$$\begin{array}{l} \underset{tarrow\infty }{\lim}\frac{B\left(t\right)}{t}=0\;a.s. \end{array}$$

Hence,


$$\begin{array}{l} \underset{tarrow\infty }{\lim\sup}\frac{\ln\;I\left(t\right)}{t}\leq \left(\tau +\mu +\sigma \right)\left({ℛ}_{0}^{S}-1\right). \end{array}$$

When $R_{0}^{S}<1$, the right-hand side is negative, implying


$$\begin{array}{l} \underset{tarrow\infty }{\lim I\left(t\right)}=0\;a.s. \end{array}$$

## 4 Numerical results

The numerical simulations presented in this study are illustrative and are not calibrated to a specific country dataset. Parameter values were selected from published pneumonia modeling studies and adjusted within biologically plausible ranges to demonstrate the qualitative dynamics of the deterministic and stochastic models. The noise intensity parameters were selected as the moderate values of environmental variability and were analyzed using sensitivity analysis to determine their effects on disease persistence and extinction. The initial conditions S(0) = 0.05, C(0) = 0.30, I(0) = 0.40, and R(0) = 0.25 are normalized proportions used for illustrative purposes in the simulations. These values do not represent actual epidemiological prevalence but are chosen to demonstrate the qualitative dynamics of the model. Because there are no closed-form solutions for the stochastic system, the numerical approximations were obtained using the Euler-Maruyama method, stochastic Runge-Kutta method, and a stochastic non-standard finite difference (NSFD) method. All simulations were conducted using MATLAB R2023a. The aim of the numerical simulation is to demonstrate stochastic threshold dynamics, noise-induced extinction, and compare the stability and consistency of the numerical methods. To complement the visual comparisons, we evaluated the numerical performance of the schemes using a reference solution computed with a sufficiently small time step. The comparison indicates that the stochastic non-standard finite difference (NSFD) method maintains better stability and smaller deviation from the reference solution compared to classical methods, particularly for larger step sizes (Table 1).

**Table 1:** Model parameters and numerical values.

Symbols	Value/per day	Source
Λ	0.5	Estimated from literature
μ	0.5	Estimated from literature
π	0.7096	(2)
β	0.515	(2)
θ	0.563	(2)
ω	0.1124	(2)
η	0.00641	(2)
δ	2 (DFE) 2.5 (EE)	Assumed (biologically plausible range)
σ	≥0	Assumed (biologically plausible range)
τ_*i*_:*i* = 1, 2, 3, 4	0–0.03	Assumed (biologically plausible range)
τ	0.641	Assumed (biologically plausible range)

### 4.1 Stochastic non-standard finite difference

This section introduces a non-standard finite-difference discretization method for the mathematical model (6). We divide the interval [0, L] into *MεN* subintervals, each with a step size of *h* = *L*/*M*. The approximate solutions for *S, C, I*, and *R* from Equation (6) are denoted as *S*^*n*^, *C*^*n*^, *I*^*n*^ and *R*^*n*^, respectively, where *n*= 0, 1, …, *N*. Following a set of predefined rules, system discretization (6) is detailed in (25).


$$\begin{array}{l} \begin{array}{l} S^{n+1}=\frac{S^{n}+h\Lambda +\eta hR^{n}+\sigma_{1}hS^{n}△B_{1}}{1+\frac{h\delta I^{n}}{N}+\frac{h\omega \delta C^{n}}{N}+\mu h} \end{array}\\ \begin{array}{l} C^{n+1}=\frac{C^{n}+\frac{h\delta \theta I^{n}S^{n}}{N}+\frac{h\omega \theta \delta C^{n}S^{n}}{N}+\sigma_{2}hC^{n}\Delta B_{2}}{\left(1+h(\mu +\beta +\pi )\right)} \end{array}\\ \begin{array}{l} I^{n+1}=\frac{I^{n}+\frac{h\delta (1-\theta )I^{n}S^{n}}{N}+\frac{h\delta \omega (1-\theta )C^{n}S^{n}}{N}+h\pi C^{n}+hI^{n}\Delta B_{3}}{\left(1+h(\tau +\mu +\sigma )\right)} \end{array}\\ \begin{array}{l} R^{n+1}=\frac{R^{n}+h\beta C^{n}+h\tau I^{n}+h\sigma_{4}R^{n}\Delta B_{4}}{\left(1+h(\mu +\eta )\right)} \end{array} \end{array}$$ (16)

To further assess the robustness of the model, a sensitivity analysis was conducted on key parameters, including the transmission rate and noise intensity coefficients. The results indicate that increases in noise intensity reduce the effective reproduction threshold and promote disease extinction, while higher transmission rates enhance persistence. These findings confirm that the qualitative behavior of the model remains consistent under reasonable parameter variations.

**Figure 2:**
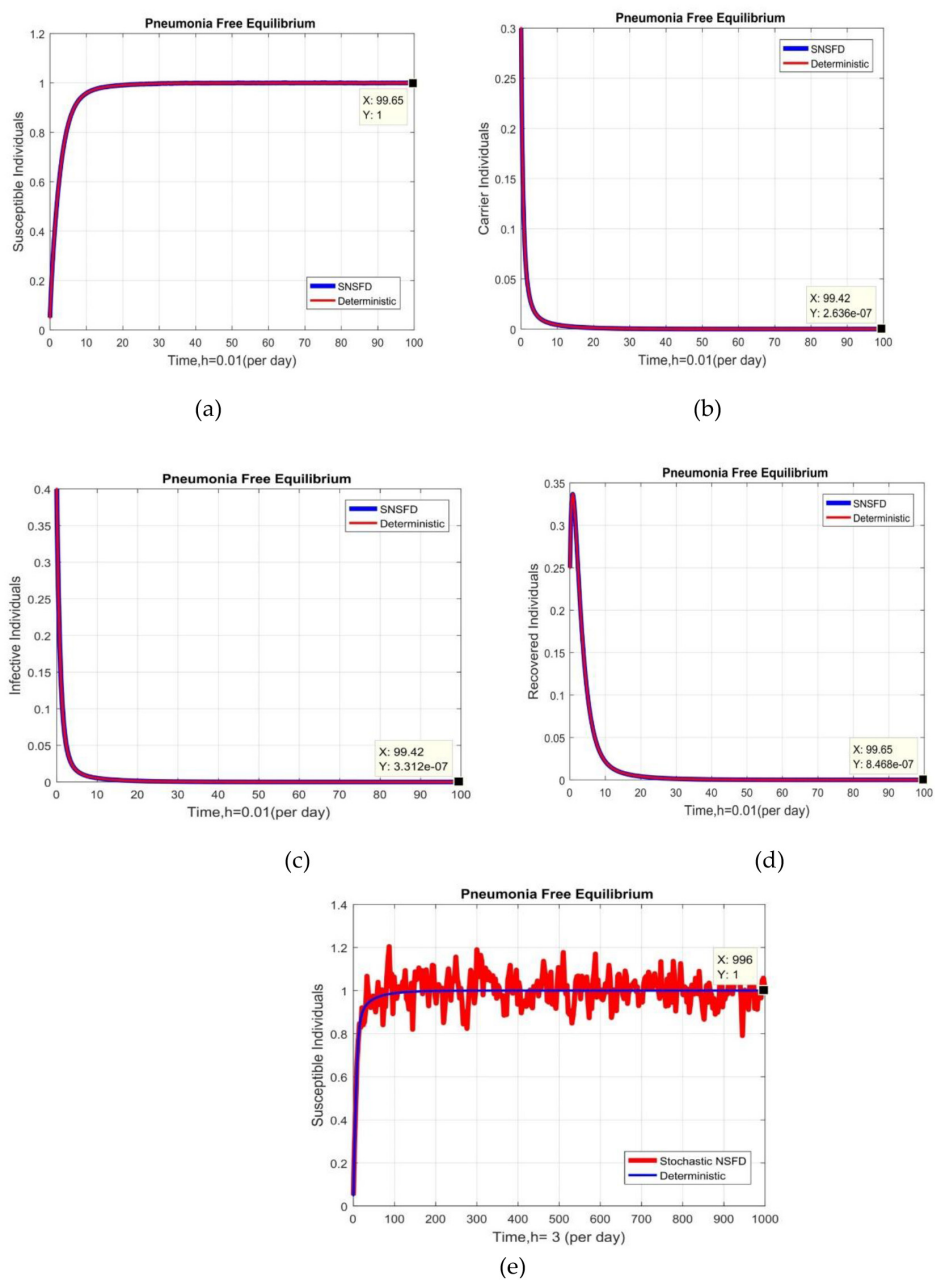
Graphical behavior of each subpopulation at pneumonia-free equilibrium **(a)** susceptible humans at h = 0.01 **(b)** Carrier humans at *h*=0.01 **(c)** Infected humans at h = 0.01 **(d)** Recovered humans at h = 0.01 **(e)** Susceptible humans at h = 20.

**Figure 3:**
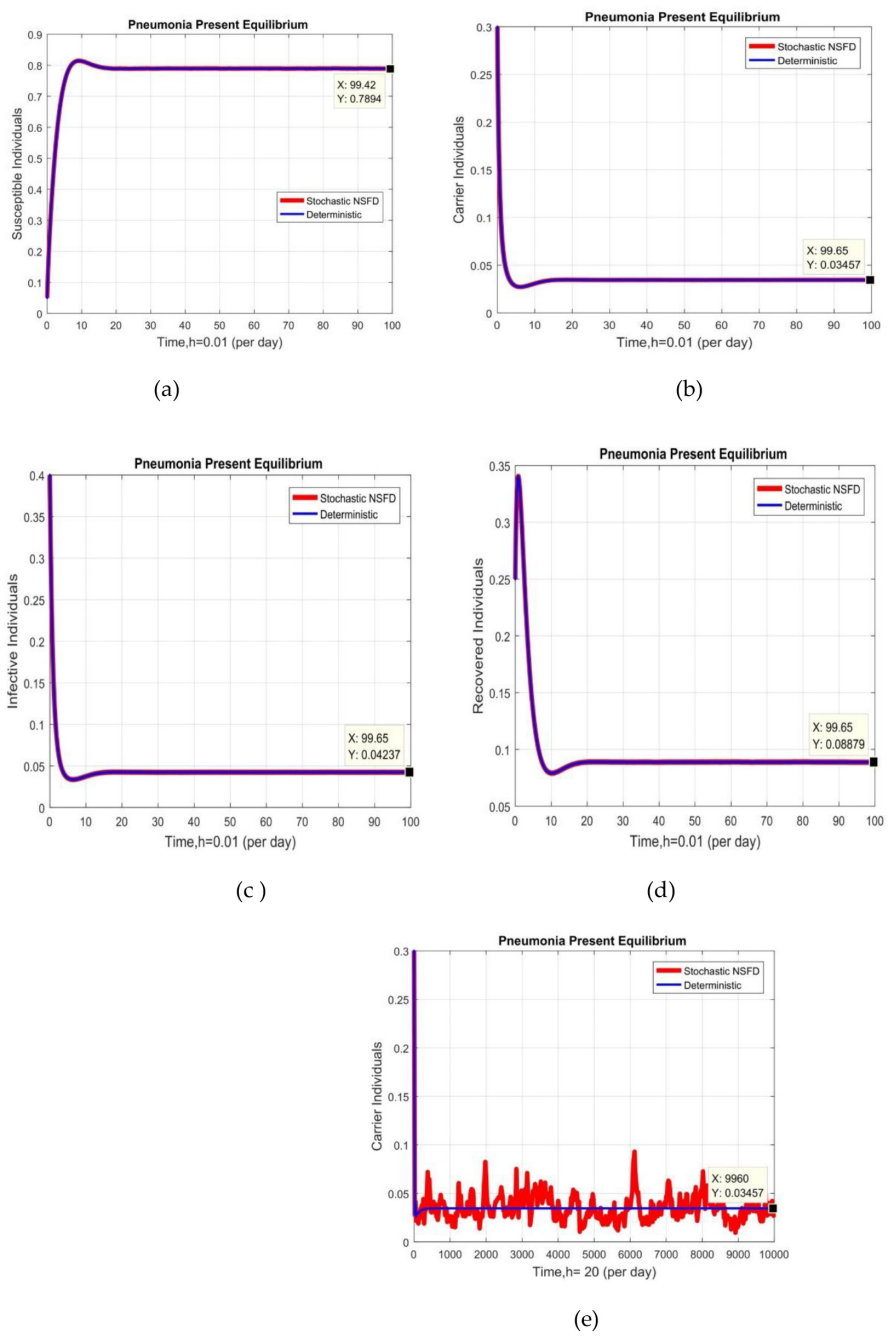
Graphical behavior of each subpopulation at pneumonia present equilibrium **(a)** susceptible humans at h = 0.01 **(b)** Carrier humans at h = 0.01 **(c)** Infected humans at h = 0.01 **(d)** Recovered humans at h = 0.01 **(e)** Carrier humans at h = 20.

**Figure 4:**
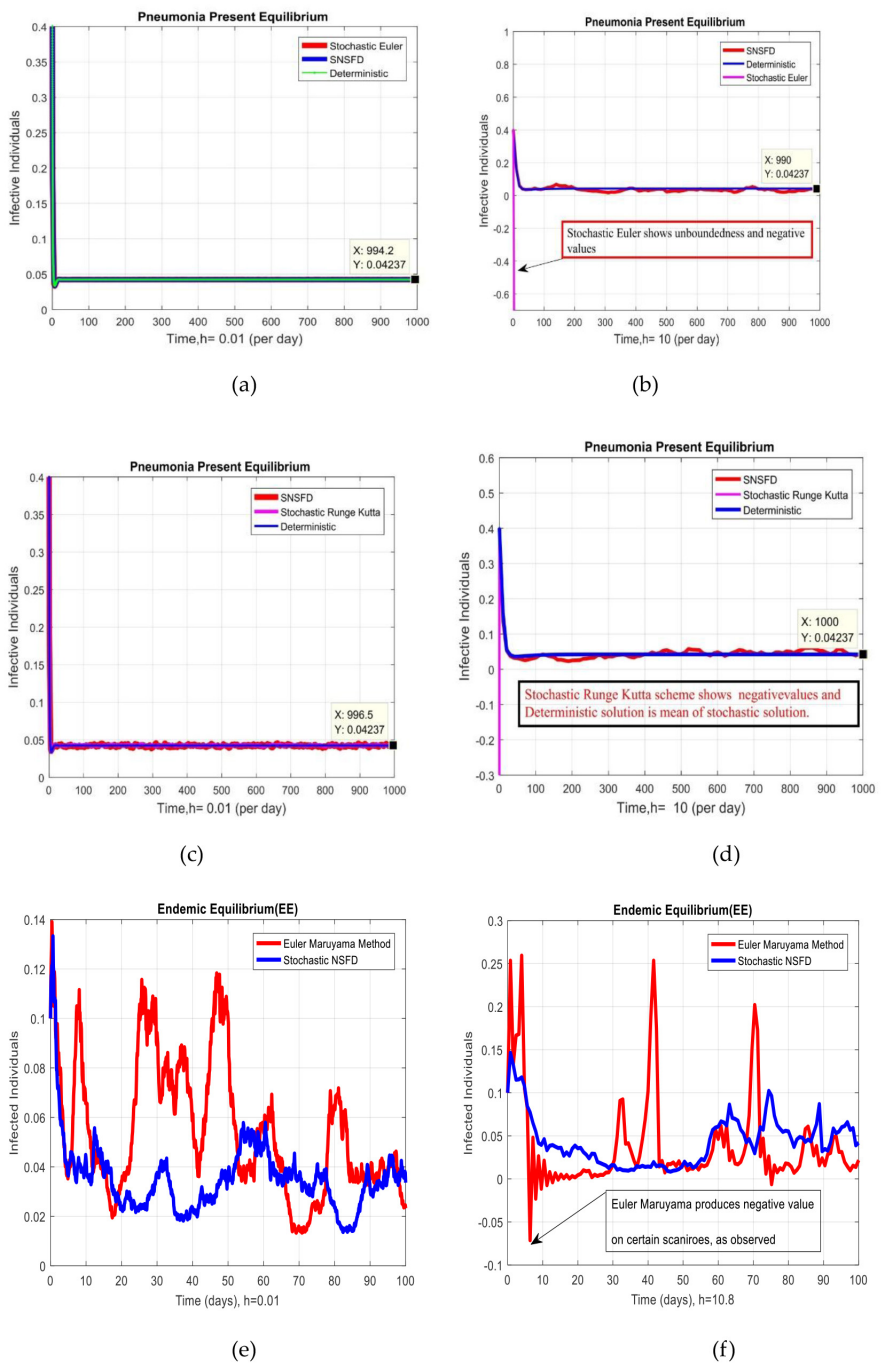
Graphical behavior of each subpopulation with a comparison of non-standard finite difference method with Euler Maruyama, Stochastic Euler, and stochastic Runge Kutta at both equilibrium **(a)** Infected humans at h = 0.01, stochastic Euler and stochastic NSFD both are convergent **(b)** Infected humans at h = 10, stochastic Euler is divergent **(c)** Infected humans at h = 0.01, stochastic Runge Kutta and stochastic NSFD both are convergent **(d)** Infected humans at h = 0.01, stochastic Runge Kutta is divergent **(e)** Infected humans at h = 0.01, Euler Maruyama and stochastic NSFD both are convergent **(f)** Infected humans at h = 10.8, Euler Maruyama is divergent.

The dynamics of the sub-populations at the disease-free equilibrium are shown in Figure 2. The simulations reveal that all sub-populations tend to the disease-free equilibrium, which verifies the stability of the equilibrium point under the selected parameters. The NSFD technique maintains positivity and stability even for large steps, but discrepancies start to arise in conventional techniques. Figure 3 depicts the presence of the disease when the system satisfies the conditions of the endemic equilibrium. The system trajectories reach a positive equilibrium point, thereby exhibiting an endemic trend. The findings emphasize the reliability of the NSFD approach in ensuring biological plausibility. The comparison of the performance of various numerical techniques is illustrated in Figure 4. It can be seen that the NSFD technique remains stable and convergent even for relatively large steps, but the Euler-Maruyama and stochastic Runge-Kutta techniques become unstable and divergent.

### 4.2 Public health implications of stochastic effects

A key contribution of this study is the identification of parameter regimes in which stochastic effects qualitatively alter pneumonia dynamics. Unlike the deterministic reproduction number *R*_0_, the stochastic reproduction threshold $R_{s}=R_{0}-\frac{\sigma^{2}}{2\left(\gamma +\mu \right)}$ explicitly shows that environmental noise reduces the effective transmission potential of the disease.

This has three important consequences:

- Lower Effective Threshold for Persistence Stochastic perturbations decrease the effective reproduction number. As the noise intensity increases, the disease persistence region shrinks.
- Noise-Induced Extinction

The parameter conditions show that *R*_0_ exceeds 1 yet $R_{0}^{S}$ remains below 1. This shows that pneumonia would continue through deterministic methods but would die out through stochastic methods. The results show that environmental changes can disrupt ongoing disease transmission. The stochastic perturbations show that effective transmission decreases when contact patterns change through intermittent distancing and behavioral changes and awareness campaigns. The stochastic framework reveals information that the deterministic model cannot show especially about how environmental changes affect threshold reduction and extinction.

#### 4.2.1 Limitations of the study

Several limitations exist for this research. The first limitation arises from the fact that the proposed mathematical model has not been calibrated using actual epidemiological data, which makes it less applicable in predicting the behavior of the disease within a specific region or community. Secondly, some of the parameters' values are not measured; rather, they have been estimated using the values obtained from previous studies. Thirdly, the assumption that the environmental fluctuations occur randomly according to Brownian motion may fail to cover other types of stochasticity. In spite of these limitations, the mathematical model has provided useful insights on the impact of environmental disturbances on disease propagation.

## 5 Concluding remarks

The research used a stochastic compartmental modeling framework to study how pneumonia transmits between different population groups. The study analyzed both deterministic and stochastic models to predict how diseases spread through four population groups: susceptible people, carriers, infected individuals, and recovered patients. The researchers proved that the model's essential characteristics which included positivity and boundedness and two types of equilibrium points existed in the model. The researchers developed a stochastic threshold condition which explained how diseases went extinct or persisted through environmental disturbances because these stochastic changes had greater impact on permanent disease outcomes than what deterministic models predicted.

Theoretical results were demonstrated through numerical simulations which showed how theoretical results work. The stochastic non-standard finite difference method produced positive bounded solutions which maintained dynamic consistency while showing better stability than the Euler-Maruyama and stochastic Euler and stochastic Runge-Kutta methods which are commonly used in stochastic numerical methods. Numerical methods which preserve structural properties of systems establish essential foundations for dependable simulations of stochastic epidemic models. From a public health perspective, the findings show that random environmental and demographic changes affect both pneumonia transmission patterns and disease persistence. The inclusion of stochastic elements in epidemic models creates a more precise representation of disease transmission which enables better evaluation of intervention methods and disease management strategies. The framework which we developed enhances understanding of pneumonia transmission patterns during uncertain conditions and serves as an effective public health decision support tool.

## Data Availability

The original contributions presented in the study are included in the article/supplementary material, further inquiries can be directed to the corresponding author.

## Footnotes

1 https://www.worldometers.info/world-population/pakistan-population/.
